# Where Opioid Overdose Patients Live Far From Treatment: Geospatial Analysis of Underserved Populations in New York State

**DOI:** 10.2196/32133

**Published:** 2022-04-12

**Authors:** Kayley Abell-Hart, Sina Rashidian, Dejun Teng, Richard N Rosenthal, Fusheng Wang

**Affiliations:** 1 Department of Biomedical Informatics Stony Brook University Stony Brook, NY United States; 2 Department of Computer Science School of Engineering and Applied Sciences Stony Brook University Stony Brook, NY United States; 3 Department of Psychiatry Stony Brook Medicine Stony Brook, NY United States

**Keywords:** opioid use disorder, opioid overdose, buprenorphine, naloxone, geospatial analysis, epidemiology, opioid pandemic, public health

## Abstract

**Background:**

Opioid addiction and overdose have a large burden of disease and mortality in New York State (NYS). The medication naloxone can reverse an overdose, and buprenorphine can treat opioid use disorder. Efforts to increase the accessibility of both medications include a naloxone standing order and a waiver program for prescribing buprenorphine outside a licensed drug treatment program. However, only a slim majority of NYS pharmacies are listed as participating in the naloxone standing order, and less than 7% of prescribers in NYS have a buprenorphine waiver. Therefore, there is a significant opportunity to increase access.

**Objective:**

Identifying the geographic regions of NYS that are farthest from resources can help target interventions to improve access to naloxone and buprenorphine. To maximize the efficiency of such efforts, we also sought to determine where these underserved regions overlap with the largest numbers of actual patients who have experienced opioid overdose.

**Methods:**

We used address data to assess the spatial distribution of naloxone pharmacies and buprenorphine prescribers. Using the home addresses of patients who had an opioid overdose, we identified geographic locations of resource deficits. We report findings at the high spatial granularity of census tracts, with some neighboring census tracts merged to preserve privacy.

**Results:**

We identified several hot spots, where many patients live far from the nearest resource of each type. The highest density of patients in areas far from naloxone pharmacies was found in eastern Broome county. For areas far from buprenorphine prescribers, we identified subregions of Oswego county and Wayne county as having a high number of potentially underserved patients.

**Conclusions:**

Although NYS is home to thousands of naloxone pharmacies and potential buprenorphine prescribers, access is not uniform. Spatial analysis revealed census tract areas that are far from resources, yet contain the residences of many patients who have experienced opioid overdose. Our findings have implications for public health decision support in NYS. Our methods for privacy can also be applied to other spatial supply-demand problems involving sensitive data.

## Introduction

Nonmedical opioid use, opioid use disorder, and opioid overdose are increasing problems in the United States. As of 2009, poisoning (mainly from drug overdose) surpassed motor vehicle accidents as the leading cause of injury-related death in US adults [1]. In 2016, the rate of opioid-related mortality alone surpassed firearms and motor vehicle accidents in the United States, killing over 42,000 people [2,3]. An estimated 350,000 people have died in the United States from causes related to opioids in the period from 1999 to 2016 [2].

The opioid epidemic has proved particularly severe in the Eastern United States [2]. Due to its large population, New York State (NYS) represents a large proportion of opioid overdose deaths nationally. In 2018, NYS had 3697 overdose deaths, the fifth highest of any state [4]. While other states in the Eastern United States successfully reduced the opioid prescribing rate between 2013 and 2017, prescribing in most NYS counties has remained steady [5]. Recent data show fatality rates continue to rise. On Long Island, the rate of opioid overdose increased by over 250% between 2010 and 2016. In Suffolk county alone, there were 365 opioid-related deaths in 2016 [3]. Because the number of people in NYS already experiencing opioid use disorder is large, and recent efforts to curtail prescribing may be insufficient, downstream approaches such as opioid use disorder treatment and emergency overdose treatment will continue to be essential.

Several papers have examined the spatial distribution of patients with opioid overdose or opioid use disorder in NYS. Epidemiological analyses are presented in Schoenfeld et al [3] with a focus on demographic factors and a high-resolution spatial analysis of the Long Island area. Similar analyses at the zip code level with statewide coverage are presented in Chen et al [6] and Xiang et al [7]. However, these papers rely on data from the NYS Department of Health Statewide Planning and Research Cooperative System (SPARCS) for admissions from 2004 through 2016. Since SPARCS has now released data through 2019, we report an updated zip code level map for opioid overdose similar to those in Chen et al [6].

With the exception of the most recent data, the spatial and demographic trends of opioid use disorder and opioid overdose are well studied, with many hot spots and risk factors identified. However, limited research is available in the literature regarding the spatial distribution of treatment resources relative to need. A few works have investigated spatial availability in specific regions, such as an analysis of naloxone deserts in New Jersey cities and an investigation of travel distances from sites of opioid overdose to medication-assisted treatment sites in Columbus, Ohio [8,9]. In this paper, we explore the availability of naloxone (for opioid overdose) and buprenorphine (for opioid use disorder) relative to the locations of the patients who may need them across the entirety of NYS.

The medication naloxone, often known by the brand name Narcan, is an opioid receptor antagonist that is highly effective at reversing an opioid overdose. Naloxone has been used in hospitals and emergency departments for four decades, and its safety and efficacy are well established [1,10-12]. If naloxone is administered before death, even by a layperson, opioid overdose survival approaches 100% [1,10]. However, availability of naloxone in medical settings alone may be insufficient. Opioid overdoses typically cause death in just 1 to 3 hours, and bystanders often do not call medical services for fear of police involvement [1,10,13,14]. Some studies found that medical services were called in less than 50% of incidents, even when bystanders had been trained on how to respond to an opioid overdose [1,13,14]. For these reasons, the World Health Organization recommends take-home naloxone, meaning that persons at risk of an opioid overdose and their household members or other contacts should carry naloxone in preparation for an emergency [15]. In 2018, the US Surgeon General echoed this advisory, emphasizing the role of family, friends, community members, and health care workers in preparing for opioid overdoses, which may involve misuse of prescription opioids, illicit opioids, and even high-dose prescriptions taken as directed [16].

To increase the availability of naloxone to laypeople, most US states have instituted open prescriptions known as standing orders [17,18]. Participating pharmacies (naloxone pharmacies) can offer this prescription to anyone who requests naloxone, circumventing the need for an individual visit with a physician and thereby reducing barriers associated with cost, time, and physician availability [17]. Standing orders have been significantly associated with increases in naloxone prescriptions and decreases in opioid-related deaths without affecting rates of nonmedical opioid use [17,19,20]. Standing orders may also accommodate lay prescribing (eg, through police officers or other community distribution programs), but the analysis by Gertner et al [17] showed that naloxone access laws specifically increased prescriptions from pharmacies, excluding lay prescriptions, suggesting an important role for naloxone pharmacies in fulfilling standing orders. However, only 2678 of more than 5000 NYS pharmacies are listed as participating in the naloxone standing order [21,22], suggesting a large opportunity to increase the impact of standing orders.

Another important class of medications are those that treat opioid use disorder itself by preventing opioid withdrawal and reducing cravings. Buprenorphine (brand names Suboxone, Subutex, etc) and methadone are first-line treatments for opioid withdrawal and maintenance therapy [23]. Unlike methadone, buprenorphine is a partial receptor agonist with a ceiling effect, meaning that additional doses beyond a threshold do not produce an increased effect. This feature renders buprenorphine safer than other full agonist opioids, an important factor since methadone overdose is a serious risk with methadone treatment [24-26]. There is mixed evidence for this difference in practice; one cross-sectional study found less mortality with buprenorphine than methadone [27]. Further, a major benefit of buprenorphine in the treatment of opioid use disorder is its high affinity for the mu receptor. This effect blocks the activity of other opioids, rendering concurrent use of other opioids generally ineffective and likely deterring further use [28]. Additionally, buprenorphine is a kappa receptor antagonist. Since the kappa receptor can cause dysphoria when stimulated, patients may tolerate buprenorphine better than methadone, which has some kappa agonist properties [26]. In addition to its safety and tolerability, buprenorphine is effective in treating opioid use disorder [29]. Meta-analyses found buprenorphine similarly or slightly less effective than methadone in retaining patients in treatment. Buprenorphine was equally effective to methadone for relieving symptoms of withdrawal [26].

Despite its effectiveness and relative safety, buprenorphine remains a schedule III controlled substance. While methadone can only be prescribed for opioid use disorder through a federally licensed treatment program, individual practitioners can prescribe buprenorphine through a waiver program in accordance with the Drug Addiction Treatment Act of 2000 [30]. Physicians must complete an 8-hour course and apply for the waiver [31]. Nurse practitioners and physician assistants are also eligible but must complete a 24-hour course [32]. Although the number of waivered prescribers in the United States has risen steadily, fewer than 5000 prescribers in NYS have obtained buprenorphine waivers despite NYS having over 77,000 licensed physicians [33]. Furthermore, the waiver program limits how many prescriptions a prescriber may have at any one time. Physicians can be licensed to prescribe to a maximum of 30, 100, or 275 patients, but the vast majority (72.7% nationally) are only certified for 30 patients [34].

Despite the development of buprenorphine waivers and standing orders for naloxone, these life-saving medications are not always accessible. For instance, a report by the US Office of the Inspector General found significant geographic disparities in buprenorphine access at the county level across the United States [35]. Our work assesses where buprenorphine and naloxone are available in NYS, finding disparities at fine spatial resolution by measuring the distance to these resources for patients who have visited a hospital for an opioid overdose. In particular, this work seeks to identify regions of insufficient resources, where opioid overdose patients live in the absence of a nearby naloxone standing order pharmacy or buprenorphine-licensed prescriber. Identification of these regions is crucial for public health efforts to bridge gaps and ensure patients have access to these essential medications. Further, our methods may serve to support other states in identifying their low-access regions for improved health policy activities.

## Methods

### Data Sets and Preprocessing Methods

For this study, we used several public data sets and one private data set. Addresses of pharmacies participating in the naloxone standing order were downloaded from the NYS Department of Health website. The data included 2678 sites, excluding one repeated record [21]. The Python library tabula-py was used to extract the data from PDF format [36]. We used the most recent issue available at the time of writing, which was last updated in September 2019.

Addresses of buprenorphine providers in NYS were downloaded from the Buprenorphine Practitioner Locator [37]. This tracking service is provided by the US Department of Health and Human Services through the Substance Abuse and Mental Health Services Administration (SAMHSA).

To identify the locations of opioid overdose patients, private data were used from SPARCS, a statewide claims database of inpatient and outpatient medical encounters. Patient home addresses were collected from encounters from 2004 to 2019 that contained one or more *International Classification of Diseases, Ninth Revision* (ICD-9) or ICD-10 codes corresponding to the SPARCS data dictionary for opioid overdoses [38]. These codes include accidental and intentional opioid poisoning by a variety of agents such as heroin, methadone, and synthetic narcotics. Although nearly all the pharmacy and prescriber addresses were usable, many of the patient addresses in SPARCS were incomplete or otherwise not able to be geocoded, such as “Homeless,” “XX,” street intersections, and post office boxes. All patient data were accessed securely and aggregated behind a firewall in accordance with our SPARCS data use agreement and our institutional review board protocol at Stony Brook University.

All 3 data sets provide location records in human-readable format (123 my-street, my-town/county my-state, 12345). For the zip code–level map of opioid overdose rates, we used the zip code from the text address, and for the county-level summaries, we used the county name in the text address. For the other mapping, aggregation, and spatial computations, we needed locations to be represented in latitude and longitude coordinates. A geocoder is a tool for converting text addresses to latitude and longitude coordinates; we used the EaserGeocoder developed by Rashidian et al [39]. This geocoder has been shown to be more accurate than other open-source geocoders and comparable to popular commercial services such as Google and MapQuest. This geocoder was chosen because it allowed us to perform all geocoding behind a firewall and without sharing data on the web, pursuant with our data use agreement and institutional review board protocol. To format the addresses for the EaserGeocoder, further data cleaning methods were applied, such as stripping text after a comma, stripping letters that occur before the first numeral in an address, removing apartment and suite numbers, and converting one to 1.

In the naloxone pharmacy and buprenorphine prescriber data sets, we manually reviewed every address that was initially rejected by the geocoder. Many were post office boxes, street intersections, or plazas. Where possible, we searched for the business or provider name and replaced the invalid address with its corresponding street address. After review, the vast majority of resource addresses were geocoded (Table 1), resulting in 2678 pharmacy locations and 4478 prescriber locations. Due to size and data sensitivity, we did not individually review the patient addresses. Of the patient addresses, 140,219 were geocoded (Table 1). We then excluded multiple encounters with the same patient identifier, keeping only the most recent encounter that was successfully geocoded. A final total of 107,493 patient locations were available for geospatial analysis.

**Table 1:** Addresses geocoded in each address data set.

	Entries for New York State, n	Rejected by EaserGeocoder, n	Number geocoded, n (%)
SAMHSA^a^ provider list	4484	6	4478 (99.87)
NYSDOH^b^ pharmacy list	2678	0	2678 (100)
SPARCS^c^ OOD^d^ patient addresses	174,484	34,295	140,219 (80.34)

^a^SAMHSA: Substance Abuse and Mental Health Services Administration.

^b^NYSDOH: New York State Department of Health.

^c^SPARCS: Statewide Planning And Research Cooperative System.

^d^OOD: opioid overdose.

### Methods for Resource Distance Analysis and Privacy Preservation

To assess resource availability, we sought to calculate the distance from patient residences to the nearest resources. In order to preserve privacy, we represented each point location by its enclosing census tract. SPARCS cell size policies prohibit reporting cells with fewer than 11 members. Therefore, tracts with 1 to 10 patients were merged with neighboring tracts until every polygon with a nonzero number of patients contained at least 11 patients. For each census tract or group of merged census tracts (MCTs), we calculated the distance from the polygon’s centroid to the nearest resource of each type. Spatial operations were performed using a PostGIS extension of a PostgreSQL database. Specifically, PostGIS was used to find the census tract that intersected with each patient point, merge census tracts, find the centroid of the newly formed MCTs, and calculate distance from each centroid to the nearest naloxone pharmacy and the nearest buprenorphine prescriber [40].

The software ArcGIS Desktop (Esri) was used to visualize data as maps. To compute density of patients far from a given resource, we first filtered the data set to include the MCTs whose centroids were >10 km (6.2 miles) from the nearest resource. This distance was chosen because it represents a relatively substantial travel distance, especially in areas with limited public transportation. Lower distance thresholds, such as 1, 2, and 4 miles, have been used in works focusing on specific urban areas such as Baltimore City, Maryland, and Columbus, Ohio [9,41]. However, research not restricted to urban areas shows that many patients travel much farther; a study of methadone patients across the United States found that 40% of patients traveled 10 or more miles to reach an opioid treatment program, and 6% traveled more than 50 miles [42]. For our statewide analysis, we chose 10 km to identify relatively underserved suburban and rural areas. After filtering for resource-far MCTs, we then used ArcGIS’s generate random points tool to generate a number of random points inside each MCT equal to the number of patients living in the MCT. Kernel density estimation was then performed on the generated points, using the kernel density tool in ArcMap with distance metric set to geodesic [43].

The programming language Python 2.7 (Python Software Foundation) was used with the library pandas for data cleaning, such as preparing the addresses for geocoding, and the Python library SQLAlchemy was used for interacting with PostGIS in Python [44].

## Results

### Zip Code–Level Rates of Opioid Overdose for 2017-2019

**Figure 1:**
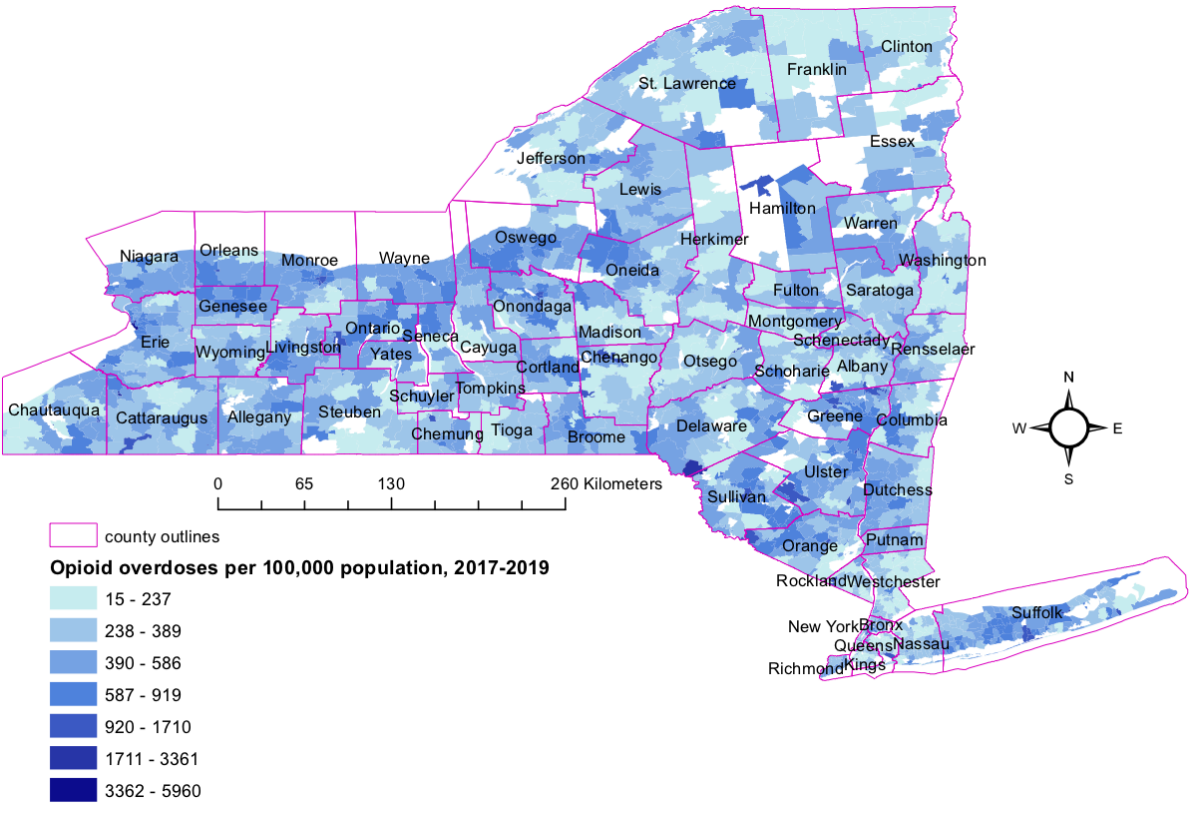
Rate of opioid overdose per 100,000 persons at the zip code tabulation area level, 2017-2019, with county outlines overlaid for reference.

First, we report an updated map of opioid overdose rates to supplement the older data published in Chen et al [6]. In this analysis, we included every opioid overdose admission with a valid zip code, without excluding multiple encounters from the same patient. Figure 1 shows the number of opioid overdose events per 100,000 residents at the zip code tabulation area level for this 3-year period. Several areas with high rates reported in Chen et al [6] continued to have high rates for recent years, such as southern central Long Island, northern Seneca county, southwestern Cattaraugus county, southern Saint Lawrence county, western Orange county, much of Greene county, and the area around the city of Buffalo. Some new hot spots are also visible, such as the area around the city of Rochester, the southwestern tip of Delaware county, and southern parts of Albany county.

If opioid overdoses were distributed randomly across zip codes, with the likelihood of each event occurring in a given zip code dependent on the zip code’s population, one would still expect to observe higher rates in some areas and lower rates in others. However, many of the zip codes were observed to have much higher rates than expected by chance, as shown in Multimedia Appendix 1, Table S1. Still, given that our primary goal was to locate high numbers of patients rather than identify underlying causes, even hot spots that occurred by chance should still be worthy of attention.

### Geospatial Analysis of Distance to Buprenorphine and Naloxone Resources

Because naloxone pharmacies and buprenorphine prescribers are not distributed evenly, lack of participation disproportionately affects certain regions of NYS. We explored this issue in respect to the locations of actual patients treated for opioid overdose in NYS.

First, we offer a summary of the number of patients and resources in each county (Multimedia Appendix 1, Table S2.) Even at this low granularity, disparities are evident. Notably, the entirety of Hamilton county was found to have zero buprenorphine-waivered prescribers and zero naloxone pharmacies. Fortunately, this remote county was home to only 21 opioid overdose patients. In the other counties, the number of patients per prescriber and patients per pharmacy varied widely. Three counties had at least 100 patients per buprenorphine-waivered prescriber (Washington, Cayuga, and Cattaraugus), while 8 counties had fewer than 25 patients per prescriber. The number of patients per naloxone pharmacy was likewise highly variable, with 4 counties having over 90 patients per pharmacy (Sullivan, Putnam, Orleans, and Allegany) and 7 counties having fewer than 40 patients per pharmacy. However, a high number of resources in a county does not necessarily indicate sufficient access for the whole county, since resources are often concentrated in certain subregions of a county.

**Figure 2:**
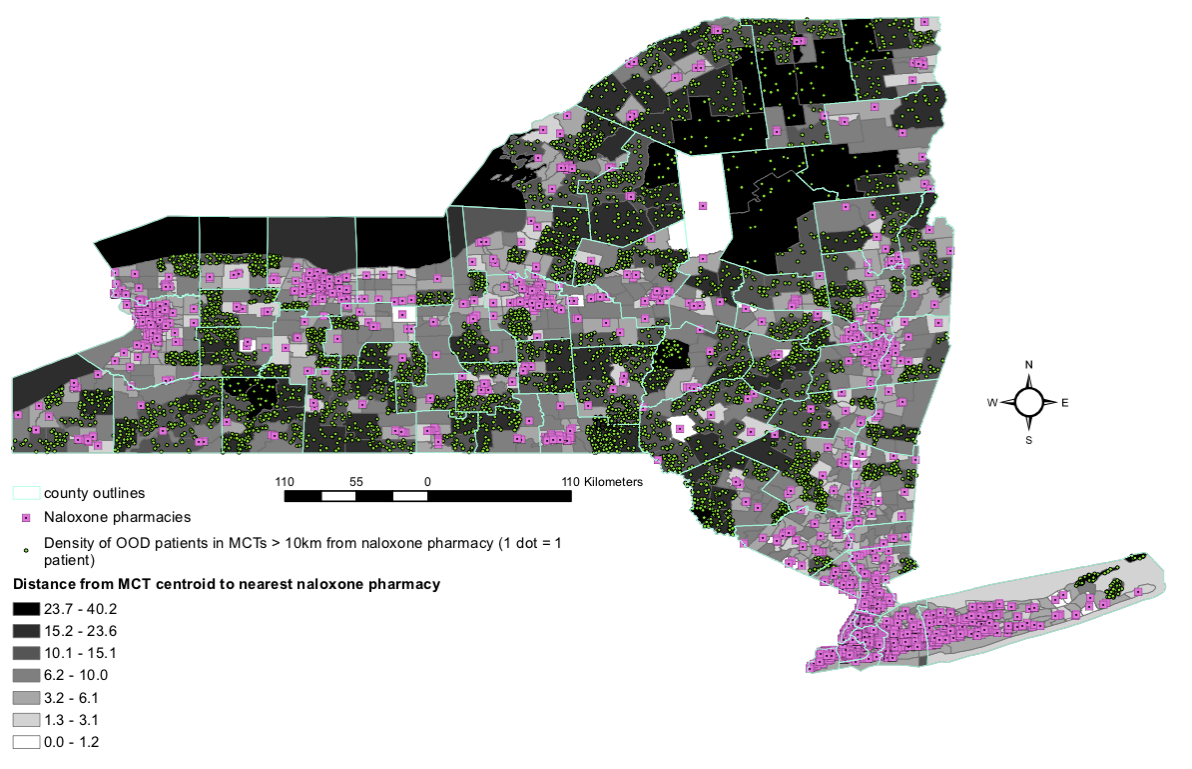
Distance in kilometers of merged census tract centroids to nearest naloxone pharmacy and density of opioid overdose patients (admissions 2004-2019) in each merged census tract. OOD: opioid overdose; MCT: merged census tract.

**Figure 3:**
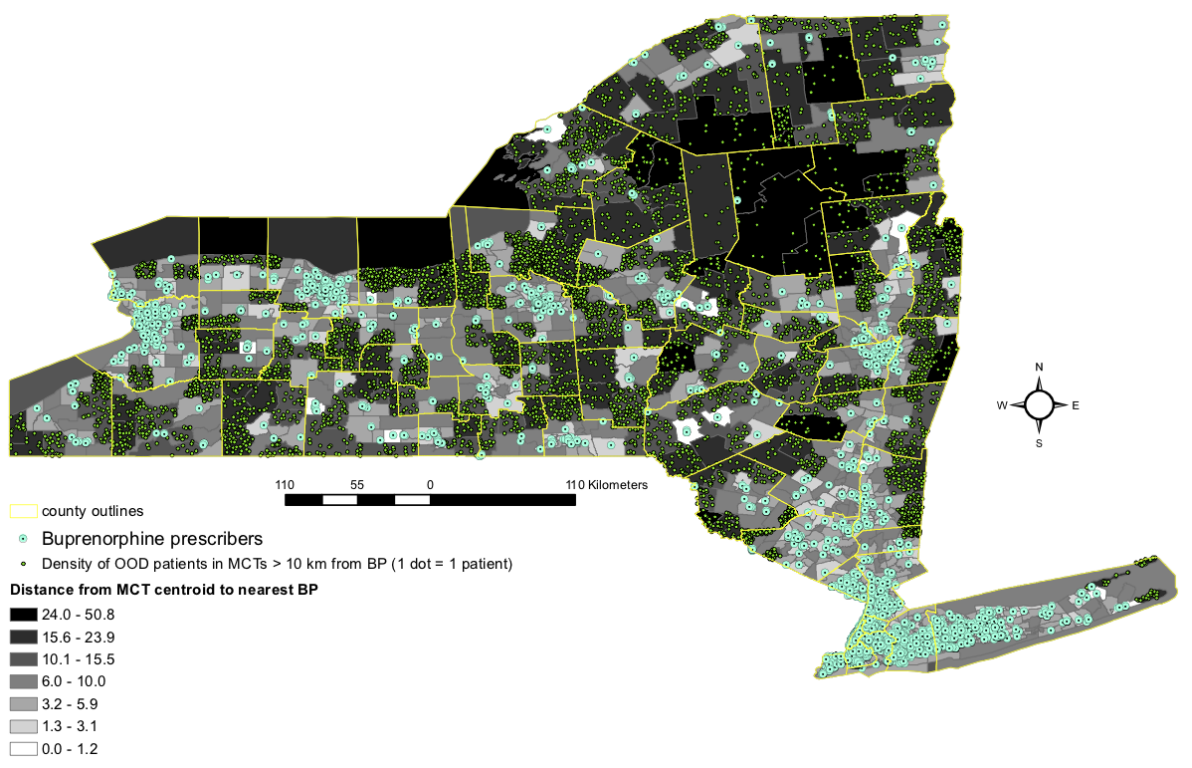
Distance in kilometers of merged census tract centroids to nearest buprenorphine-waivered prescriber and density of opioid overdose patients (admissions 2004-2019) in each merged census tract. OOD: opioid overdose; MCT: merged census tract; BP: buprenorphine prescriber.

In order to find subregions with unmet needs, we present a supply and demand analysis at the high spatial resolution of the MCT. We visualized distance to resources as choropleths showing distance of MCTs to nearest resources. Figure 2 shows the distance from each MCT centroid to the nearest naloxone pharmacy, and Figure 3 shows the distance from each MCT centroid to the nearest buprenorphine provider. In both maps, a dot-density tool shows the number of patients living in the MCTs >10 km from the nearest resource.

The naloxone pharmacy map shows that most pharmacies are clustered in the urban and suburban areas, including Long Island. Naloxone pharmacies are absent in much of the northern part of the state, in the southwest, and in several of the south central areas. Although Long Island has a large number of pharmacies, its easternmost tip is lacking this resource. As for the number of patients whose residences overlap with these areas, the southwestern and south central areas appear most salient. Like the naloxone map, the buprenorphine prescriber map shows most prescribers are in urban and suburban areas, with a deficit on the eastern tip of Long Island. A large area without coverage appears in the northern central part of the state, and smaller areas appear along the border of Lake Ontario (in Oswego, Cayuga, and Wayne counties), along the southern border, and in the southern central part of the state (around Chenango and Cortland counties). Overlapping patients appear most numerous in Wayne and Oswego counties. However, in both maps, visual identification of the densest areas is limited by the fact that the dots obscure each other in dense areas. For this reason, we directly calculate and visualize density in the next section.

### Kernel Density Estimation Reveals Hot Spots of High Need Relative to Resources

**Figure 4:**
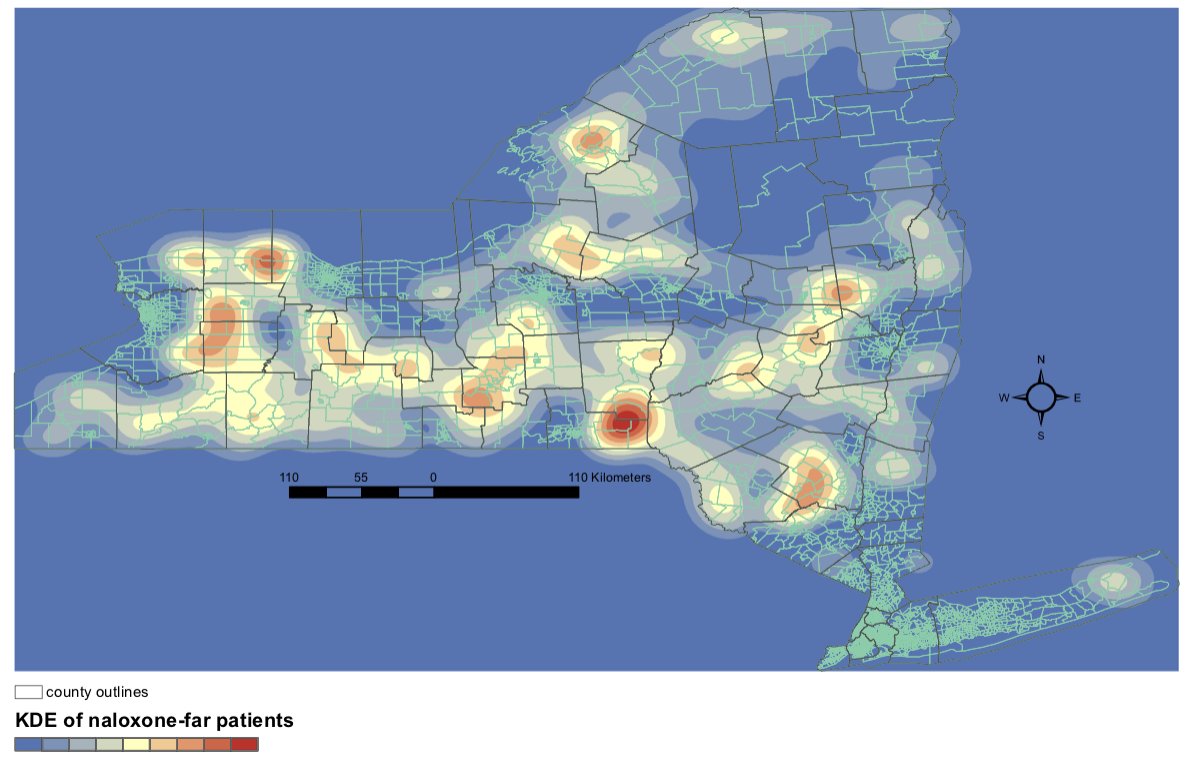
Density of opioid overdose patients living in merged census tracts >10 km from the nearest naloxone pharmacy in New York State. KDE: kernel density estimation.

**Figure 5:**
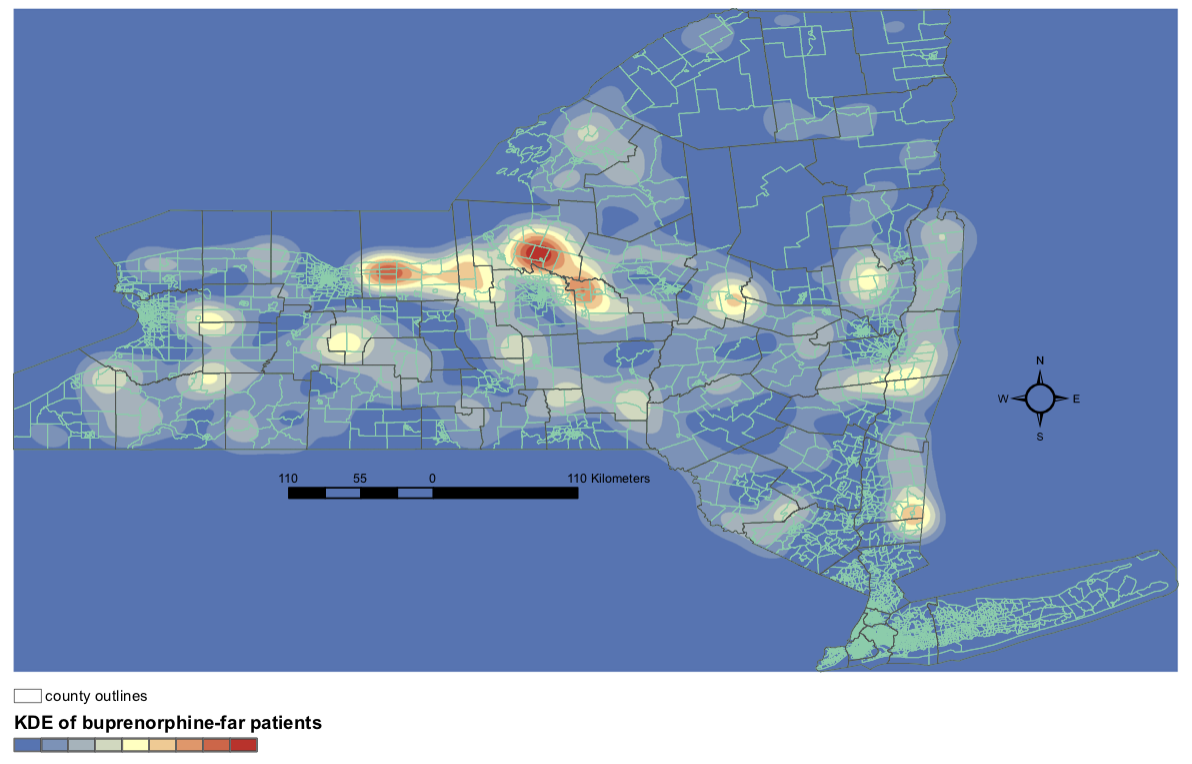
Density of opioid overdose patients living in merged census tracts >10 km from the nearest New York prescriber with a buprenorphine waiver. KDE: kernel density estimation.

Figures 2 and 3 highlight regions of NYS that are far from opioid use disorder and opioid overdose treatment resources but do not fully reflect patient density in these regions. To examine density of resource-far patients, we performed kernel density estimation. We visualized the density of patients living in MCTs >10 km from the nearest naloxone pharmacy or >10 km from the nearest buprenorphine-waivered prescriber (Figure 4 and Figure 5, respectively). For naloxone distance, the highest density region was found in eastern Broome county (Figure 4). Although a number of naloxone pharmacies exist in Broome county, they are all in the western part of the county, mostly near the city of Binghamton. For buprenorphine distances, the densest regions were in the northwestern part of the state, particularly Oswego and Wayne counties (Figure 5). Together, these data outline significant geographic disparities existing in targetable subregions of NYS.

## Discussion

### Summary and Comparison With Related Studies

Our updated map shows that opioid overdose continues to be frequent across NYS, with particular burden in certain areas. Our analysis of treatment resource locations shows that access to naloxone and buprenorphine is far from universal. In Broome county and several other regions, areas without nearby naloxone pharmacies overlap with the residences of a large number of opioid overdose patients. In regions such as Oswego county, areas without buprenorphine providers likewise overlap with the residences of many patients. These findings have a variety of implications for targeted interventions.

Our analysis of new SPARCS data (2017-2019) provides an update to the existing research detailing opioid overdose epidemiology in NYS [3,6]. Past studies have examined naloxone and buprenorphine resources using a variety of methods, often focusing on a small geographic area. Lozo et al [8] investigated pharmacies’ participation in naloxone programs in 10 cities in New Jersey in conjunction with the rate of opioid-related hospital visits. Hyder et al [9] calculated distances from the sites of opioid overdose events in Columbus, Ohio, to the nearest medication for opioid use disorder treatment sites. Guerrero and Kao [45] examined the relationship between integrated treatment providers for substance abuse and neighborhood demographics such as income and race in Los Angeles county, California. Our work provides an important new contribution, assessing both naloxone and buprenorphine resources across a large geographic area.

### Study Interpretation and Implications

It is not surprising for rural areas to have reduced proximity to resources that are often concentrated in municipal areas. However, our findings show that the overlap of patients with low-resource areas is not a uniform phenomenon across rural regions; instead, hot spots exist in particular subregions. We hope that the identification of these regions will help public health agencies to prioritize them through targeted interventions.

In the case of naloxone, it may be possible to recruit pharmacies that do not yet participate in the naloxone standing order. However, some areas might lack any pharmacies, with or without naloxone. A Google Maps search for pharmacy in the area of the Broome county hot spot shows one nearby pharmacy, on the border of Broome and Delaware counties, in the town of Deposit. This pharmacy is not listed in the directory of naloxone pharmacies, so its absence is not a geocoding error. It may be an ideal candidate for recruitment, yet having naloxone on standing order at a single pharmacy in the area might still be insufficient, and some other hot spots might not have any nearby pharmacies. One far-reaching solution is to make naloxone available over the counter (OTC), meaning that it could be stocked in any store and not limited to pharmacies [12]. The US Food and Drug Administration (FDA) has already promoted OTC naloxone as an essential step for improving naloxone accessibility. When potential manufacturers cited OTC labeling requirements as a barrier, the FDA developed a prototype label and completed their own comprehensibility testing, essentially greenlighting the process in 2018 [46,47]. Given the continued lack of OTC products, there may be other barriers or simply a lack of financial interest for pharmaceutical companies; in this case, a government contract could bridge the gap.

As for buprenorphine, it may likewise be possible to recruit prescribers in the regions we identified. However, training clinicians and obtaining waivers does not necessarily mean that they will be able or willing to accept new patients. It may be preferable to expand psychiatric care resources in general, perhaps incentivizing the establishment of new practices in these areas, and even incentivizing young professionals to enter addiction psychiatry. Telehealth modalities are also an important route for improved care access, especially in remote areas. However, the federal government requires completion of an in-person physical to initiate buprenorphine treatment. This requirement was temporarily lifted during the COVID-19 pandemic in order to reduce in-office visits, leading to multiple successful telehealth programs [48-50]. Simply making this change permanent could improve buprenorphine accessibility for patients in the regions we identified.

Extremely remote areas represent a further challenge. Remote areas with low numbers of patients potentially spread over a large geographic area (eg, Herkimer and Hamilton counties) may be difficult to reach with spatially targeted interventions. These patients further underscore the need for telehealth prescribing, mail delivery of prescriptions, and nonpharmacy naloxone, as well as improved case finding and community outreach strategies.

Finally, spatial availability of resources such as naloxone and buprenorphine is only one of many obstacles to overdose prevention and recovery. The opioid epidemic is a complex crisis with many drivers; the spatial decision support suggested by this work is in no way intended as a sole solution. Other important resource types include methadone, naltrexone, fentanyl testing strips, therapy, and peer support. Given that social determinants of health play a significant role in the risk for opioid use disorder and opioid overdose, novel public education, identification, and engagement strategies might be implemented differentially targeting the described “landscapes of despair” that likely overlap with the census tracts underresourced with naloxone and medication for opioid use disorder prescribers [51]. Further, programs and policies must work to address the widespread financial and emotional distress that has worsened during the COVID-19 pandemic.

### Limitations

For this study, we processed a large amount of real-world data. These data provide a powerful picture of opioid resource need and availability. However, we faced several limitations in working with these data. Even after intensive data cleaning, about 20% of patient addresses in SPARCS were not able to be geocoded. This problem might disproportionately affect certain populations, particularly homeless patients. In theory, a homeless patient should still have their address recorded as the place they reside, such as the address of a homeless shelter, park, or street corner. However, many addresses were recorded for opioid overdose patients in SPARCS such as undomiciled or homeless shelter, making geocoding impossible. This limitation is especially important given the increased risk of overdose in homeless individuals [52]. Our analyses are also unable to count opioid overdose patients who did not go to a hospital, which could be even more common for rural patients.

A further limitation of SPARCS data is the inevitable temporal lag; SPARCS data takes time to be compiled and released, so analyses cannot reflect the latest trends. In the Overdose Detection Mapping Application Program (ODMAP), overdoses are reported by first responders in a mobile app so that they can be compiled in almost real time [53]. The ODMAP website shows that there are participating agencies in every New York county. However, this fact does not necessarily indicate complete coverage of overdose events, since a single county has many agencies that would all need to participate. For example, the ODMAP website only lists one participating police department in Broome county, the City of Binghamton Police Department. Government agencies that have access to these data should use them to complement the less current but more complete analyses like the ones presented in this work.

The data for prescriber and pharmacy locations present some unique challenges as well. One limitation is that because NYS data were used, low-resource areas might appear exaggerated near the borders of the state. In particular, a high-density region of patients far from buprenorphine providers appears on the eastern border north of New York City (Figure 5), but these patients may be able to access resources across the border in Danbury, Connecticut, depending on their insurance coverage. Although the Broome county hot spot occurs near the southern border of NYS, it borders on a low-density region of Pennsylvania, so this still appears to be a high-need area. An important complication of prescriber data is that some prescribers work at multiple locations but do not always register every location in SAMHSA’s buprenorphine locator. It is also unknown how many NYS buprenorphine prescribers chose not be listed publicly in SAMHSA’s directory. One analysis of administrative records found that just over half of waivered prescribers had chosen to be listed publicly, although the number could be higher in NYS [54]. However, unlisted providers may be more difficult for prospective patients to find, since they are not in the directory, and unlisted providers may be less likely to be accepting new opioid use disorder patients. Therefore, gaps in the availability of listed providers may still represent important resource deficits.

Additionally, geocoding is not 100% accurate; even commercial geocoders such as Google and MapQuest only agree on about 95% of test addresses [39]. Geocoding error explains why a buprenorphine prescriber appeared in Hamilton county, when the county-level summary of prescribers showed zero buprenorphine prescribers in Hamilton county. Because the MCTs surrounding this erroneous point are so large, it so happens that their centroids are still >10 km away, so this point did not materially affect our results.

Last, our privatization methods necessarily introduce some error. For example, it is possible to have an MCT whose centroid is far from resources, even though parts of the MCT are not, especially for large or unusually shaped MCTs. If most patients actually live in the part of the MCT that is nearer to resources, a misleading hot spot could appear. To privately address the possibility of misleading hot spots, we provide close-up maps of the regions we highlighted, showing the boundaries of each MCT shape, the associated patient counts, and the nearby resources (Multimedia Appendix 2-4). Ideally, one would calculate the distance from each patient address coordinate to the nearest resource instead of generalizing their location to the MCT. Such analysis may be feasible for public health planning operations that have access to these data. However, due to the possibility of reverse geocoding, such results cannot be made public.

### Conclusions

Geospatial analysis of naloxone and buprenorphine resources revealed areas of need across NYS. The locations of these subregions should be informative to other researchers and to NYS health agencies. Rather than trying to provide for all remote areas, public health efforts can prioritize these subregions and reach high-need patients. In addition, our approach may be helpful to other states in identifying targets for resource application to address their opioid epidemic with more local efficiency. It may also be applicable to other spatial resource problems involving sensitive data.

## Appendix

Multimedia Appendix 1 Supplementary tables.

Multimedia Appendix 2 Close-up of Broome county and surrounding areas, with merged census tract polygons labeled with the number of patients in that polygon. Pink lines show original census tract boundaries that were removed during merging.

Multimedia Appendix 3 Close-up of western New York State, with merged census tract polygons labeled with the number of patients in that polygon. Pink lines show original census tract boundaries that were removed during merging.

Multimedia Appendix 4 Close-up of northwestern New York State, with merged census tract polygons labeled with the number of patients in that polygon. Pink lines show original census tract boundaries that were removed during merging.

## Abbreviations

FDA: Food and Drug Administration
ICD-9: International Classification of Diseases, Ninth Revision
MCT: merged census tract
NYS: New York State
ODMAP: Overdose Detection Mapping Application Program
OTC: over-the-counter
SAMHSA: Substance Abuse and Mental Health Services Administration
SPARCS: Statewide Planning And Research Cooperative System
