# Supplementary material for: Where Opioid Overdose Patients Live Far From Treatment: Geospatial Analysis of Underserved Populations in New York State
Source: JMIR Public Health Surveill. 2022 Apr 12;8(4):e32133. doi: 10.2196/32133 (PMC9044159; doi:10.2196/32133)
Supplement: Multimedia Appendix 1 [file publichealth_v8i4e32133_app1.docx]

**Multimedia Appendix X.** Supplementary tables.

**Table S1.** OOD rate of top 30 ZIP codes (with population > 300)

| ZIP code | OOD events | Population | rate per 100,000 | P value |
| --- | --- | --- | --- | --- |
| 14203 | 73 | 1618 | 4512 | < 0.0001 |
| 14604 | 47 | 1743 | 2697 | < 0.0001 |
| 11947 | 9 | 416 | 2163 | < 0.0001 |
| 12760 | 9 | 482 | 1867 | 0.00014 |
| 13202 | 93 | 5438 | 1710 | < 0.0001 |
| 14202 | 60 | 3911 | 1534 | < 0.0001 |
| 13103 | 5 | 326 | 1534 | 0.00884 |
| 14720 | 10 | 675 | 1481 | 0.00035 |
| 14605 | 168 | 12610 | 1332 | < 0.0001 |
| 14480 | 11 | 833 | 1321 | 0.00042 |
| 11951 | 180 | 13680 | 1316 | < 0.0001 |
| 12120 | 8 | 613 | 1305 | 0.0027 |
| 10519 | 4 | 316 | 1266 | 0.03347 |
| 12456 | 8 | 639 | 1252 | 0.00361 |
| 14621 | 416 | 33802 | 1231 | < 0.0001 |
| 12780 | 28 | 2312 | 1211 | < 0.0001 |
| 11950 | 187 | 16268 | 1149 | < 0.0001 |
| 14061 | 4 | 349 | 1146 | 0.04569 |
| 14613 | 168 | 14730 | 1141 | < 0.0001 |
| 12475 | 4 | 354 | 1130 | 0.04589 |
| 13832 | 8 | 709 | 1128 | 0.00627 |
| 13203 | 173 | 16029 | 1079 | < 0.0001 |
| 10035 | 356 | 33969 | 1048 | < 0.0001 |
| 14471 | 28 | 2672 | 1048 | < 0.0001 |
| 12432 | 5 | 492 | 1016 | 0.0404 |
| 12565 | 15 | 1487 | 1009 | 0.00079 |
| 12428 | 66 | 6602 | 1000 | < 0.0001 |
| 14608 | 122 | 12268 | 994 | < 0.0001 |
| 14872 | 6 | 605 | 992 | 0.02906 |
| 12172 | 4 | 405 | 988 | 0.07184 |

Among ZIP codes with a population of at least 300, the top thirty ZIP codes by rate of OOD per capita are shown.

P values were estimated by randomly assigning the 73,187 OOD events to a given ZIP code with probability equal to the population of the ZIP code divided by the total population of New York State, and counting the number of times (out of 100,000 samples) that the number of simulated events was equal to or greater than the actual observed number.

**Table S2**. Patient and resource summary by county

| County | OOD patients | | Buprenorphine prescribers | Naloxone pharmacies | Patients per prescriber | Patients per pharmacy |
| --- | --- | --- | --- | --- | --- | --- |
| Albany | 2018 | | 84 | 49 | 24 | 41 |
| Allegany | 364 | | 5 | 4 | 73 | 91 |
| Bronx | 10943 | | 376 | 231 | 29 | 47 |
| Broome | 2059 | | 32 | 35 | 64 | 59 |
| Cattaraugus | 599 | | 6 | 7 | 100 | 86 |
| Cayuga | 654 | | 6 | 11 | 109 | 59 |
| Chautauqua | 1119 | | 24 | 19 | 47 | 59 |
| Chemung | 721 | | 17 | 11 | 42 | 66 |
| Chenango | 372 | | 10 | 5 | 37 | 74 |
| Clinton | 468 | | 18 | 14 | 26 | 33 |
| Columbia | 573 | | 6 | 9 | 96 | 64 |
| Cortland | 411 | | 8 | 8 | 51 | 51 |
| Delaware | 359 | | 10 | 6 | 36 | 60 |
| Dutchess | 2582 | | 54 | 36 | 48 | 72 |
| Erie | 9641 | | 243 | 133 | 40 | 72 |
| Essex | 204 | | 10 | 6 | 20 | 34 |
| Franklin | 267 | | 9 | 8 | 30 | 33 |
| Fulton | 413 | | 7 | 7 | 59 | 59 |
| Genesee | 616 | | 15 | 8 | 41 | 77 |
| Greene | 415 | | 10 | 10 | 42 | 42 |
| Hamilton | 21 | | 0 | 0 | NA | NA |
| Herkimer | 440 | | 5 | 8 | 88 | 55 |
| Jefferson | 774 | | 23 | 19 | 34 | 41 |
| Kings | 11255 | | 430 | 377 | 26 | 30 |
| Lewis | 151 | | 4 | 2 | 38 | 76 |
| Livingston | 503 | | 11 | 6 | 46 | 84 |
| Madison | 539 | | 7 | 11 | 77 | 49 |
| Monroe | 6396 | | 190 | 106 | 34 | 60 |
| Montgomery | 489 | | 9 | 8 | 54 | 61 |
| Nassau | 7772 | | 275 | 168 | 28 | 46 |
| New York | 9551 | | 948 | 316 | 10 | 30 |
| Niagara | 2394 | | 33 | 31 | 73 | 77 |
| Oneida | 2047 | | 42 | 29 | 49 | 71 |
| Onondaga | 4647 | | 109 | 78 | 43 | 60 |
| Ontario | 971 | | 25 | 15 | 39 | 65 |
| Orange | 2969 | | 71 | 46 | 42 | 65 |
| Orleans | 392 | | 5 | 4 | 78 | 98 |
| Oswego | 1159 | | 13 | 17 | 89 | 68 |
| Otsego | 416 | | 21 | 8 | 20 | 52 |
| Putnam | 692 | | 12 | 7 | 58 | 99 |
| Queens | | 7876 | 261 | 194 | 30 | 41 |
| Rensselaer | | 1084 | 16 | 22 | 68 | 49 |
| Richmond | | 3671 | 124 | 75 | 30 | 49 |
| Rockland | | 1212 | 61 | 30 | 20 | 40 |
| Saint Lawrence | | 741 | 31 | 21 | 24 | 35 |
| Saratoga | | 1296 | 35 | 28 | 37 | 46 |
| Schenectady | | 1244 | 34 | 24 | 37 | 52 |
| Schoharie | | 177 | 8 | 4 | 22 | 44 |
| Schuyler | | 113 | 2 | 2 | 57 | 57 |
| Seneca | | 288 | 5 | 4 | 58 | 72 |
| Steuben | | 699 | 18 | 11 | 39 | 64 |
| Suffolk | | 13725 | 359 | 195 | 38 | 70 |
| Sullivan | 833 | | 14 | 6 | 60 | 139 |
| Tioga | 232 | | 6 | 4 | 39 | 58 |
| Tompkins | 581 | | 25 | 13 | 23 | 45 |
| Ulster | 1630 | | 40 | 19 | 41 | 86 |
| Warren | 549 | | 13 | 17 | 42 | 32 |
| Washington | 445 | | 4 | 8 | 111 | 56 |
| Wayne | 744 | | 8 | 13 | 93 | 57 |
| Westchester | 4593 | | 175 | 108 | 26 | 43 |
| Wyoming | 310 | | 10 | 4 | 31 | 78 |
| Yates | 192 | | 3 | 3 | 64 | 64 |

Patients were counted in the county of the home address given at their most recent hospital visit for opioid overdose in SPARCS.

OOD: Opioid overdose
