# Supplementary figures and images for: Where Opioid Overdose Patients Live Far From Treatment: Geospatial Analysis of Underserved Populations in New York State
Source: JMIR Public Health Surveill. 2022 Apr 12;8(4):e32133. doi: 10.2196/32133 (PMC9044159; doi:10.2196/32133)

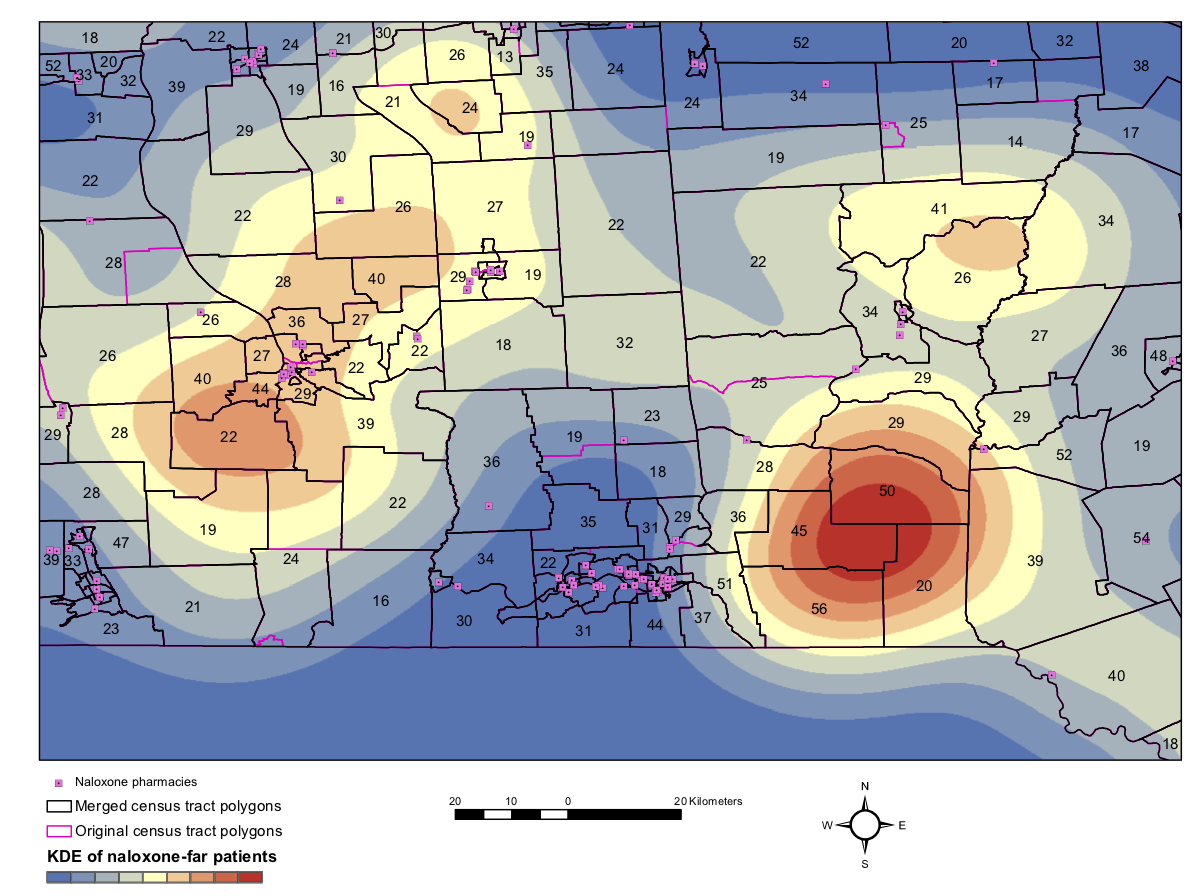

Supplement: Multimedia Appendix 2 [file publichealth_v8i4e32133_app2.png]

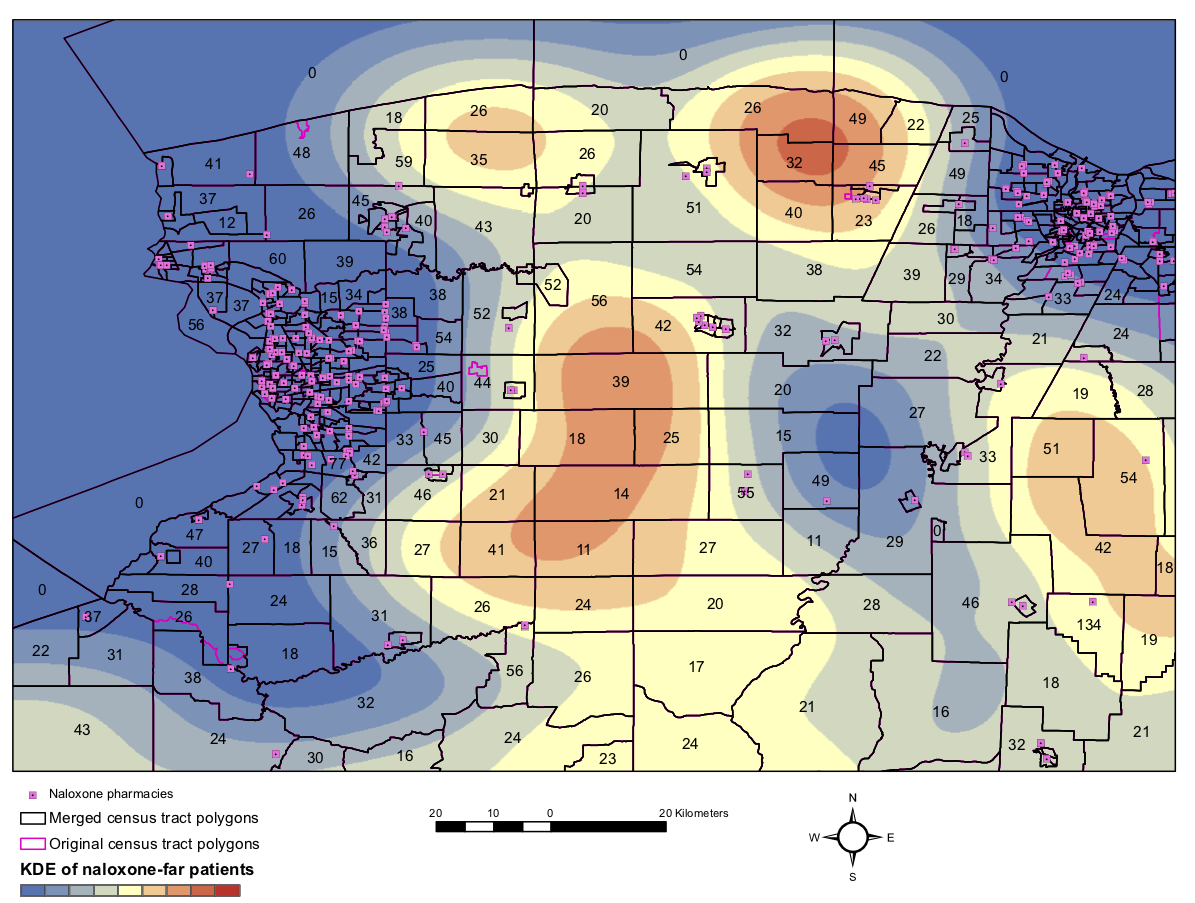

Supplement: Multimedia Appendix 3 [file publichealth_v8i4e32133_app3.png]

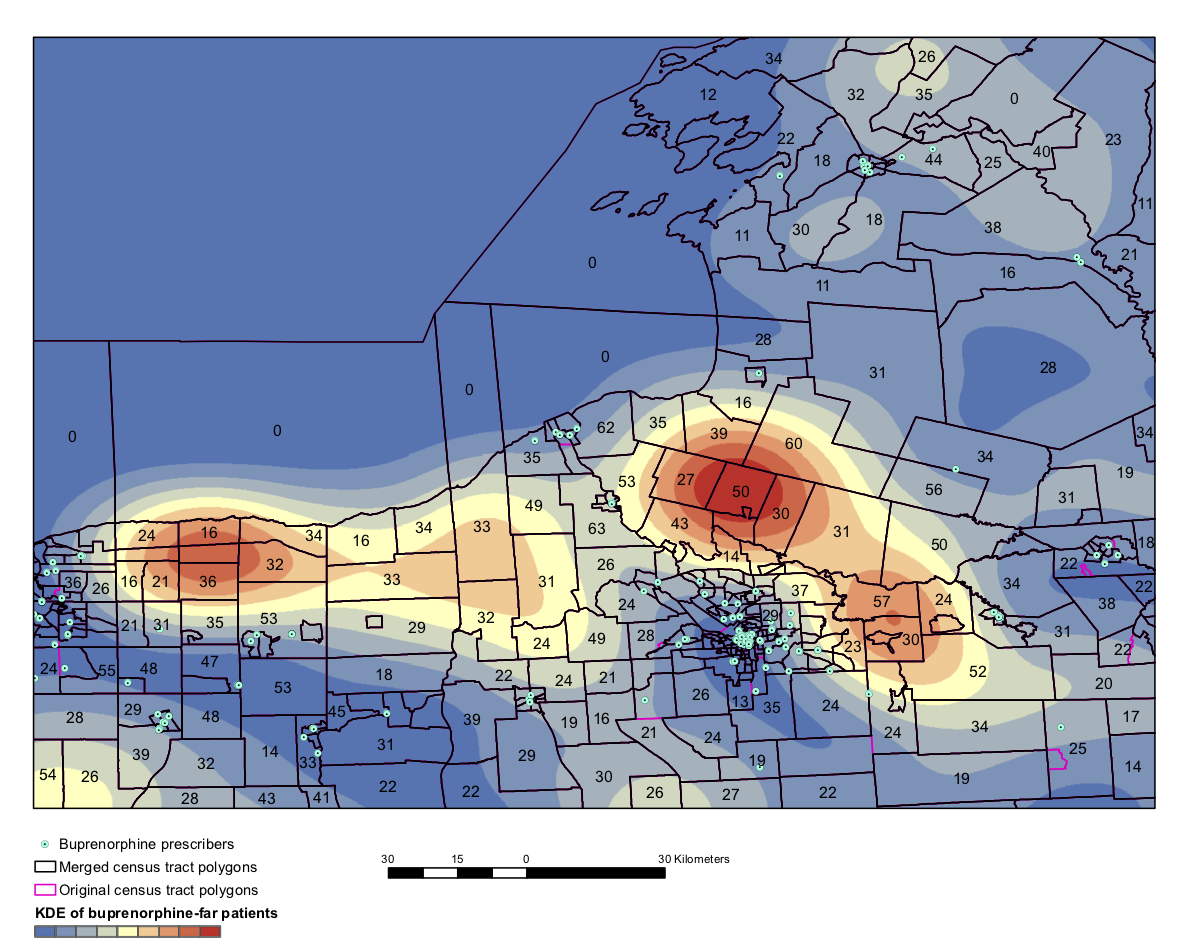

Supplement: Multimedia Appendix 4 [file publichealth_v8i4e32133_app4.png]
